# Unmet needs after prevention: a model for managing premature surgical menopause post-risk-reducing bilateral salpingo-oophorectomy

**DOI:** 10.1016/j.gore.2025.101963

**Published:** 2025-10-05

**Authors:** Sara Perelmuter, Laura Keenahan, Alicia Mecklai, Panagiota Andreopoulou, Sangeeta Kashyap, Jamieson Greenwald, Lisa Mosconi, Michael Battista, Ravi N. Sharaf, Melissa K. Frey

**Affiliations:** a Weill Cornell Medical College, New York, NY, United States; b Weill Cornell Medicine, Department of Obstetrics and Gynecology, New York, NY, United States; c Weill Cornell Medicine, Division of Cardiology, Department of Medicine, New York, NY, United States; d Weill Cornell Medicine, Division of Endocrinology, Department of Medicine, New York, NY, United States; e Weill Cornell Medicine, Division of Neuroscience, Department of Neurology and Radiology, New York, NY, United States; f Weill Cornell Medicine, Division of Gynecologic Oncology, Department of Obstetrics and Gynecology, New York, NY, United States; g Weill Cornell Medicine, Genetics and Personalized Cancer Prevention Program, New York, NY, United States; h Weill Cornell Medicine, Division of Gastroenterology, Department of Medicine, New York, NY, United States

**Keywords:** Premature Surgical Menopause, Risk-Reducing Salpingo-Oophorectomy, Cancer Prevention, Cardiovascular Disease Risk, Cognitive Decline, Osteoporosis Prevention

## Abstract

Risk-reducing bilateral salpingo-oophorectomy (rrBSO) is a standard of care for individuals with BRCA1/2 pathogenic variants and other hereditary ovarian cancer syndromes. While the oncologic benefits are clear, the abrupt estrogen deprivation of premature surgical menopause confers increased risks of cardiovascular disease, osteoporosis, and cognitive decline—yet long-term preventive care remains inconsistently provided. This commentary presents a multidisciplinary pilot model developed at a major academic center to bridge this gap.

Eligible patients seen in a gynecologic oncology clinic received an educational flyer summarizing chronic disease risks and evidence-based prevention strategies. Referral algorithms were created to streamline access to cardiology, endocrinology, and neurology.

From December 2023 to October 2024, 49 patients (median age 49 years, range 27–61) engaged with the program. All patients accepted the flyers, and at one-month follow-up (47 reached), 98% reported positive feedback, 94% found the material accessible, and 23% noted increased motivation for preventive care. The most frequent comment was that the flyers were easily understandable (70%). Eight patients were referred to preventive cardiology (6 scheduled), and six to endocrinology for low bone density (5 scheduled).

These preliminary findings suggest that patients with premature surgical menopause are highly motivated to engage in chronic disease prevention when provided with accessible education and a clear pathway to care. Integrating survivorship-focused preventive strategies into routine gynecologic oncology visits is feasible, well-received, and addresses a pressing unmet need. As cancer prevention improves, the next frontier must include structured care for long-term health after premature surgical menopause.

## Highlights

• Pilot program addresses chronic disease risk post-risk-reducing oophorectomy.
• Patients report improved awareness of CVD, osteoporosis, and dementia risks.
• 96% of patients engaged in follow-up; most found the material accessible.
• Preventive care integration into oncology workflow shown to be feasible.
• Multidisciplinary approach bridges cancer care with long-term health needs.

## 1 Introduction

Risk-reducing bilateral salpingo-oophorectomy (rrBSO) is a cornerstone of cancer prevention for individuals with hereditary ovarian cancer syndromes. Recent studies suggest that 0.5% of the general population carries a BRCA1/2 pathogenic variant (PV)(Manickam et al., 2018), with prevalence as high as 2.5% among individuals of Ashkenazi Jewish ancestry(Gabai-Kapara et al., 2014). Guidelines recommend that all women with BRCA1/2 PVs undergo rrBSO. While the oncologic benefits of rrBSO are well established, a growing body of evidence underscores the significant non-oncologic consequences of premature surgical menopause, including increased risks of cardiovascular disease, osteoporosis, and neurological diseases such as Alzheimer’s disease, dementia, and Parkinsonism(Shuster et al., 2008, Parker et al., 2013, Rocca et al., 2022). These adverse outcomes are largely attributed to the abrupt and early loss of endogenous hormones. In the general population, premature surgical menopause has been associated with elevated all-cause and cause-specific mortality, particularly among women who undergo rrBSO prior to age 45 years and do not receive hormone replacement therapy(Parker et al., 2013, Rivera et al., 2009, Gierach et al., 2014). Studies suggest that approximately 40–70% of women with BRCA1/2 PVs proceed with risk-reducing surgery(Lamacki et al., 2024, Lim et al., 2021, Park et al., 2020, Zimovjanova et al., 2023), underscoring both the current and projected future burden of premature surgical menopause in this population.

Despite the well-documented association between premature surgical menopause and adverse long-term health outcomes, preventive care following rrBSO remains underutilized and is not routinely integrated into standard post-surgical management. The predominant clinical emphasis on cancer risk reduction may inadvertently neglect opportunities to address and mitigate the elevated risks of chronic conditions such as cardiovascular, bone, and neurologic diseases. In response, we developed and implemented a multidisciplinary pilot program aimed at incorporating evidence-based strategies for cardiovascular disease, osteoporosis, and Alzheimer’s disease risk reduction into the routine care of patients with hereditary ovarian cancer syndrome experiencing premature surgical menopause.

## 2 Program overview and rationale

**Fig. 1:**
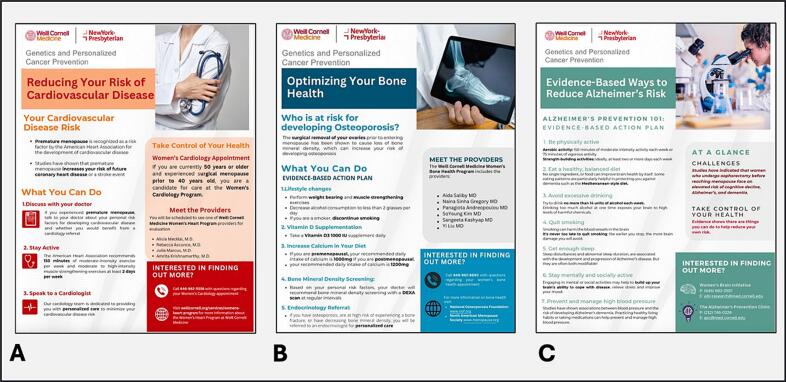
Information sheets. Cardiovascular disease (A); Bone health (B); Alzheimer’s risk (C).

As part of a quality improvement initiative, stakeholder groups were assembled including patients, patient advocates, researchers, clinical staff, and clinicians with expertise in gynecologic oncology, cancer genetics, cardiovascular disease, metabolic bone disease, and neurologic disease.. Patients with hereditary ovarian cancer syndrome and premature surgical menopause were given informational flyers containing evidence-based information on risk and preventive strategies related to cardiovascular disease, osteoporosis, and Alzheimer’s disease (Fig. 1). Based on stakeholder meetings with clinical specialists, algorithms were defined to determine patient populations with significant risk to warrant referral to specialty services and pre-established clinics: 1) Patients were referred to an endocrinology clinic if they were found on bone density imaging (DXA) to have low bone density or osteoporosis. 2) Patients were referred to a preventative cardiology clinic if they were 50 years or older with menopause at 40 years or younger, or if patients had other risk factors for cardiac disease. 3) Patients were referred to an Alzheimer’s disease / memory clinic if they had any memory complaints or concerns for dementia.

The clinical approach was informed by extensive literature demonstrating the systemic impact of early estrogen deprivation. For instance, women undergoing oophorectomy before natural menopause show significantly increased risks of cognitive impairment and dementia, with greater effects at younger ages(Rocca et al., 2007, Georgakis and Petridou, 2021). Similar trends are observed in bone metabolism, where rrBSO results in accelerated bone mineral density loss compared to natural menopause(Jiang et al., 2021). Additionally, large cohort studies, such as the Nurses’ Health Study and the Mayo Clinic Cohort, have confirmed an increased incidence of coronary heart disease and mortality in women undergoing early oophorectomy(Rivera et al., 2009, Thao et al., 2025, Rocca et al., 2016).

**Fig. 2:**
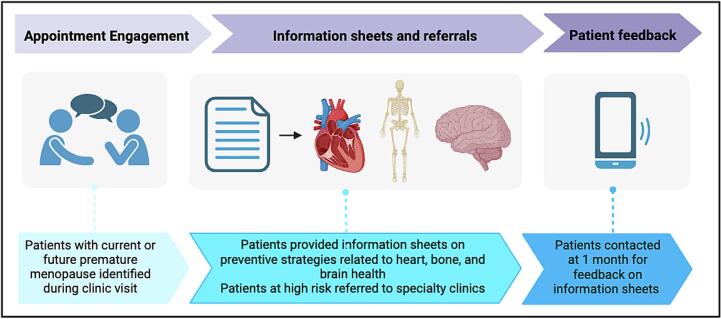
Workflow of Preventive Care Program, Speciality Referral, and Feedback Model for Patients with Premature Surgical Menopause. *Figure created by Biorender.

Patients were identified through attendance at an outpatient gynecologic oncology clinic between December 2023 and October 2024. Eligibility was based on having undergone, or being considered/scheduled to undergo, premature surgical menopause through rrBSO. Informational flyers were offered directly to patients during clinic visits. All patients that accepted the information flyers were contacted by telephone at one month to review their experience with the flyers (Fig. 2). No standard menopausal symptom management information is typically given to patients. Besides referral to speciality clinics, information on attendance or follow up duration was beyond the scope of this pilot study.

## 3 Preliminary findings and feasibility

Since initiation, the program engaged 49 eligible patients. Median patient age was 49 years (range 27–61); race included White (45, 92%), Asian (3; 6%), and Black (1; 2%); ethnicity included Non-hispanic (46; 94%) and Hispanic (3; 6%). All patients had either completed or were planning to undergo rrBSO due to hereditary cancer risk. All patients (49, 100%) offered the information flyers expressed interest and accepted the flyers. Forty-seven (96%) patients were reached for a one-month follow-up telephone call and among this group, 46 (98%) gave positive feedback on the information flyers. Eight patients had been referred to a preventative cardiology clinic, and six (75%) had scheduled an appointment at time of contact. Six patients were referred to an endocrinology clinic due to low bone densitiy and five (83%) had scheduled an appointment at time of contact. An additional seven patients had schedueled an appointment for a DXA scan based on clinical counselling and reinforced information on the flyer.

The majority (44, 94%) of patients described the information provided by the flyers as accessible, and several (11, 23%) noted increased motivation to pursue preventive care. The most common feedback was that the information flyers offered easily understandable information (33; 70%), followed by satisfaction with the content (31; 66%), the ability to take the information home (17; 36%), assistance in finding resources (11; 23%), encouragement to seek further care (8; 17%), a positive emotional response to the information (5; 11%).

Our preliminary findings support both the feasibility and acceptability of integrating preventive care into the clinical workflow of a gynecologic oncology clinic. Importantly, they also highlight a demand for information and referrals among this population. The educational component was particularly well received, suggesting that patient-centered tools may help bridge the gap between oncologic and general preventive care.

## 4 Clinical and public health implications

This initiative underscores the need for a broader conceptualization of survivorship care in patients undergoing rrBSO. The preventive management of cardiovascular disease, osteoporosis, and neurologoic disease should be considered an essential extension of cancer preventative care, particularly for individuals who undergo rrBSO at a young age. Our findings suggest that multidisciplinary collaboration is both feasible, well received by patients, and able to be implemented without substantial structural changes to existing clinical models.

## 5 Limitations and considerations

While the outcomes of our pilot program are promising, several limitations warrant consideration. Referral to specialty clinics was based on algorithms developed by stakeholder consensus, rather than validated clinical pathways. Future efforts should focus on developing and evaluating evidence-based referral algorithms to optimize care delivery and standardization. Additionally, future programs should integrate the primary care provider into this preventative care pathway. This was a single-center study with a relatively small sample size and short follow-up duration. Patient feedback was obtained via telephone survey and may be subject to recall bias and social desirability bias. In addition, while the use of informational flyers is a pragmatic approach, future studies should consider more robust, multimedia educational strategies and assess knowledge retention and behavioral change over time.

Moving forward, evidence-based and structured referral criteria are needed to determine when speciality care is indicated. Electronic health record-based prompts may further streamline referral processes and reduce variability in care delivery. Longitudinal studies are needed to assess the long-term impact of such interventions on patient outcomes, including adherence to preventive care, health-related quality of life, and disease incidence.

A further limitation relates to the broader applicability of this program to other centers. Our institution had access to specialty services in cardiology, endocrinology, and neurology, which may not be readily available in all settings. Implementation in centers with limited resources will require adaptation, including close reliance on primary care physicians to manage preventive strategies for cardiovascular disease, metabolic bone health, and cognitive decline. Strengthening the role of primary care providers in survivorship care and fostering shared decision-making between oncology and primary care will be critical to ensuring equitable delivery of preventive care. Tailoring interventions to resource availability, while maintaining patient-centeredness, will be an essential step toward broader dissemination.

## 6 Next steps in implementation

The next phase of implementation should focus on scaling this model beyond a single institution. This may involve developing streamlined referral criteria that can be adapted across a variety of practice settings, including community-based clinics with limited specialty resources. Embedding electronic health record prompts to facilitate referrals, expanding eligibility criteria, and incorporating primary care engagement will support sustainability. Additionally, educational materials could be expanded into digital and interactive formats to improve accessibility and knowledge retention.

Longitudinal evaluation of patient outcomes, including preventive care uptake, quality of life, and long-term incidence of cardiovascular, endocrine, and neurologic diseases, will be critical to establishing the value of this program. Collaboration with health systems and policymakers to advocate for resource allocation and guideline development may also accelerate widespread adoption. By integrating survivorship and preventative care into standard post-rrBSO management, this program has the potential to reshape long-term outcomes for women with hereditary ovarian cancer syndromes.

## 7 Conclusion

As the field of gynecologic oncology continues to evolve toward personalized and survivorship-focused care, it is imperative to address the full spectrum of health risks faced by women undergoing rrBSO. Our pilot study demonstrates that integrating preventive care for cardiovascular disease, osteoporosis, and neurologic disease into routine gynecologic oncology practice may be feasible and well received by patients. A multidisciplinary approach that includes cardiology, neurology, and endocrinology should be considered best practice in managing the long-term health of patients with premature surgical menopause. Future efforts should aim to standardize referral pathways, expand eligibility criteria, and evaluate the long-term benefits of such integrative care models.

## CRediT authorship contribution statement

**Sara Perelmuter:** Writing – review & editing, Writing – original draft, Visualization, Validation, Project administration, Methodology, Investigation, Formal analysis, Data curation, Conceptualization. **Laura Keenahan:** Writing – review & editing, Visualization, Validation, Project administration, Conceptualization. **Alicia Mecklai:** Writing – review & editing, Resources, Methodology, Conceptualization. **Panagiota Andreopoulou:** Writing – review & editing, Resources, Methodology, Conceptualization. **Sangeeta Kashyap:** Writing – review & editing, Resources, Methodology, Conceptualization. **Jamieson Greenwald:** Writing – review & editing, Resources, Methodology, Conceptualization. **Lisa Mosconi:** Validation, Resources. **Michael Battista:** Writing – review & editing, Resources, Methodology, Conceptualization. **Ravi N. Sharaf:** Writing – review & editing, Visualization, Resources, Project administration, Methodology. **Melissa K. Frey:** Writing – review & editing, Visualization, Validation, Supervision, Resources, Project administration, Methodology, Investigation, Formal analysis, Data curation, Conceptualization.

## Declaration of competing interest

The authors declare that they have no known competing financial interests or personal relationships that could have appeared to influence the work reported in this paper.

## References

[b0005] Manickam K., Buchanan A.H., Schwartz M.L.B. (2018). Exome sequencing-based screening for BRCA1/2 expected pathogenic variants among adult biobank participants. JAMA Netw. Open.

[b0010] Gabai-Kapara E., Lahad A., Kaufman B. (2014). Population-based screening for breast and ovarian cancer risk due to BRCA1 and BRCA2. PNAS.

[b0015] Shuster L.T., Gostout B.S., Grossardt B.R., Rocca W.A. (2008). Prophylactic oophorectomy in premenopausal women and long-term health. Menopause Int..

[b0020] Parker W.H., Feskanich D., Broder M.S. (2013). Long-term mortality associated with oophorectomy compared with ovarian conservation in the nurses’ health study. Obstet. Gynecol..

[b0025] Rocca W.A., Smith C.Y., Gazzuola Rocca L., Savica R., Mielke M.M. (2022). Association of premenopausal bilateral oophorectomy with parkinsonism and Parkinson disease. JAMA Netw. Open.

[b0030] Rivera C.M., Grossardt B.R., Rhodes D.J. (2009). Increased cardiovascular mortality following early bilateral oophorectomy. Menopause.

[b0035] Gierach G.L., Pfeiffer R.M., Patel D.A. (2014). Long-term overall and disease-specific mortality associated with benign gynecologic surgery performed at different ages. Menopause.

[b0040] Lamacki A.J., Spychalska S., Maga T. (2024). Risk-reducing salpingo-oophorectomy among diverse patients with BRCA mutations at an urban public hospital: a mixed methods study. BMJ Open.

[b0045] Lim H., Kim S.I., Hyun S., Lee G.B., Seol A., Lee M. (2021). Uptake rate of risk-reducing salpingo-oophorectomy and surgical outcomes of female germline BRCA1/2 mutation carriers: a retrospective cohort study. Yonsei Med. J..

[b0050] Young P.S., Kim Y., Kim S. (2020). Factors associated with the decision to undergo risk-reducing salpingo-oophorectomy among women at high risk for hereditary breast and ovarian cancer: a systematic review. Korean J. Women Health Nurs..

[b0055] Zimovjanova M., Bielcikova Z., Miskovicova M. (2023). Uptake and effectiveness of risk-reducing surgeries in unaffected female BRCA1 and BRCA2 carriers: a single institution experience in the Czech Republic. Cancers.

[b0060] Rocca W.A., Bower J.H., Maraganore D.M. (2007). Increased risk of cognitive impairment or dementia in women who underwent oophorectomy before menopause. Neurology.

[b0065] Georgakis M.K., Petridou E.T. (2021). Long-term risk of cognitive impairment and dementia following bilateral oophorectomy in premenopausal women—time to rethink policies?. JAMA Netw. Open.

[b0070] Jiang H., Robinson D.L., Lee P.V.S. (2021). Loss of bone density and bone strength following premenopausal risk-reducing bilateral salpingo-oophorectomy: a prospective controlled study (WHAM Study). Osteoporos Int..

[b0075] Thao, V., Borah, B., Stewart, E.A., et al., Cardiovascular disease after hysterectomy in the nurses’ health study and nurses’ health study II. Obstet Gynecol. Published online May 8, 2025. doi:10.1097/AOG.0000000000005902. 10.1097/AOG.0000000000006058 41100879

[b0080] Rocca W.A., Gazzuola-Rocca L., Smith C.Y. (2016). Accelerated accumulation of multimorbidity after bilateral oophorectomy: a population-based cohort study. Mayo Clin. Proc..

